# Semantic knowledge of words is necessary to produce an incidental self-reference effect

**DOI:** 10.3758/s13423-026-02901-y

**Published:** 2026-03-26

**Authors:** Harrison A. Paff, Kyungmi Kim, Josephine Ross, Natasha Matthews, Ada Kritkikos

**Affiliations:** 1 Psychology, Faculty of Health, University of Dundee, Scrymgeour Building, Nethergate, Dundee, DD1 4HN UK; 2 Department of Psychology, Wesleyan University, Middletown, CT USA; 3 School of Psychology, University of Queensland, Brisbane, Queensland Australia

**Keywords:** Self-reference effect, Self-related processing, Incidental processing, Word category

## Abstract

Encoding information in relation to the self produces a memory advantage compared with other encoding methods. Termed the *self-reference effect* (SRE), this self-memory advantage has typically been demonstrated when people explicitly evaluate the self-relevancy of to-be-remembered target stimuli. Notably, recent works showed that simply indicating the location of target stimuli in relation to a self-relevant versus other-relevant cue (e.g., one’s own or another person’s name) under a non-evaluative, non-referential encoding context produces a self-memory advantage. What accounts for this incidental SRE (iSRE)? In the current study, by varying the semantic properties of target stimuli, we asked whether the iSRE mainly results from (a) spontaneous evaluative self-referencing, (b) spontaneous semantic self-referencing, or (c) perceptual processing of target stimuli in spatio-temporal relation to a self-relevant cue. During encoding, participants indicated whether trait adjectives, concrete nouns, or pseudowords were presented to the left or right of their own, their friend’s, or a stranger’s name. In a subsequent source memory test, we found better source memory for words paired with one’s own name versus their friend’s and a stranger’s name for trait adjectives and concrete nouns but not for pseudowords, with the magnitude of the iSRE similar for trait adjectives and concrete nouns. No self-memory advantage with pseudowords suggests that perceptual processing of target stimuli in relation to a self-cue is insufficient to produce an iSRE, but rather the iSRE draws on a basic semantic connection between the self and a simultaneously presented target stimulus. Potential mechanisms underlying the iSRE, implications, and future research directions are discussed.

**Supplementary Information:**

The online version contains supplementary material available at 10.3758/s13423-026-02901-y.

## Introduction

In traditional self-reference effect (SRE) paradigms, participants evaluate whether trait adjectives describe themselves or another, often a celebrity (e.g., “are you [is Angelina Jolie] *confident*?”). Subsequently, self-referenced words are remembered better than other-referenced words (Klein & Loftus, 1988; Rogers et al., 1977). This evaluative SRE (eSRE) is thought to arise from superior organisation and elaboration of items bound with the self-concept relative to concepts of other people. Although item memory (memory for the information itself) is the most commonly used eSRE measure (Symons & Johnson, 1997), only source memory (memory for contextual details of the information; Durbin et al., 2017; Serbun et al., 2011) can reveal whether information is linked to the self through episodic binding (e.g., recalling that a word was encoded in reference to the self vs. a celebrity; Johnson et al., 1993). eSREs are typically larger for source than for item memory (Hamami et al., 2011; Zhang et al., 2020).

Further studies have investigated how the self modulates memory via non-evaluative task-directed associations with stimuli (Cunningham et al., 2008; Turk et al., 2008). In Turk et al. (2008), participants viewed a trait adjective appearing above or below their own or another person’s name or face (i.e., the self-cue or other-cue), and judged if the word described the depicted person (evaluative encoding) or if it appeared above the cue (incidental encoding). The eSRE was replicated, but importantly, in the incidental encoding condition, recognition was better for self- than other-paired words, suggesting that task-directed evaluative self-appraisal is not needed to produce an SRE. Termed the *incidental SRE* (iSRE), this self-memory advantage is suggested to occur due to spontaneous preferential attention to self-cues versus other-cues which enhances encoding of stimuli co-occurring with self-cues (Cunningham et al., 2014; Turk et al., 2008). The iSRE has been replicated in item and source memory using trait adjectives with young adults (Kim et al., 2018, 2019) and using pictorial concrete objects with children (Cunningham et al., 2014; Hutchison et al., 2021; Ross et al., 2011, 2020). However, the mechanism underpinning the iSRE is not established.

Although relatively few studies have investigated mechanisms underlying the iSRE, two recent ones (Kim et al., 2018, 2019) suggest an association between a target stimulus and a self-cue occurring above conscious awareness. Kim et al. (2018) showed that the iSRE in item memory for trait adjectives arises when referent names are presented above conscious awareness, with no forward or backward masks, but not below conscious awareness, with forward and backward masks. Next, Kim et al. (2019) showed that encoding a target stimulus’ spatial location in *relation* to a self-cue is necessary to produce an iSRE. They presented trait adjectives in red or green font, and participants indicated either the target stimulus font colour (non-relational encoding: “is the word colour *red* or *green*?”) or location in relation to the self- or other-cue (relational encoding: “is the word *above or below the name*?”). Only relational encoding produced an iSRE, suggesting a task-context-dependent association between target stimuli and self-cues is necessary to produce an iSRE, while a mere co-presentation of target stimuli with a self-cue is not sufficient. Collectively, Kim et al.’s (2018, 2019) findings suggest the iSRE is not likely produced solely from early, automatic attention processing, instead requiring a combination of conscious concurrent encoding of target stimuli and self-cues. Therefore, although the iSRE methodology does not require evaluation of target stimulus self-relevance, and the magnitude is smaller for iSRE than eSRE (Turk et al., 2008), spontaneous evaluative self-referencing is not ruled out as underpinning the iSRE.

If linking target stimulus semantic properties to the self may contribute to the iSRE, its presence and magnitude should depend on the degree of spontaneous evaluative self-referencing (e.g., “*agreeable* appeared on the left of my name, and I think I AM agreeable”). In contrast, if perceptual processing of target stimuli in spatio-temporal relation to a self-cue mainly contributes to the iSRE (e.g., “*agreeable* appeared on the left of MY NAME”), varying the semantic properties of target stimuli should not affect its presence/magnitude. We therefore sought to identify whether spontaneous evaluative self-referencing or enhanced perceptual processing of target stimuli in relation to a self-cue underpins the iSRE by investigating how processing three different word types of varying semantic properties (trait adjectives, concrete nouns, or pseudowords) incidentally to the self, one’s best friend, and a stranger may affect subsequent source memory for target words.

To our knowledge, no study has investigated how different word types affect iSRE presentation. Although iSREs have been found for trait adjectives (Kim et al., 2018, 2019; Kim & Philipps, 2025; Turk et al., 2008) and for pictorial concrete nouns (Cunningham et al., 2014; Hutchison et al., 2021; Ross et al., 2020), iSREs for trait adjectives and concrete nouns have never been directly compared. Interestingly, eSRE literature investigating self-other comparisons with person referents has shown that encoding concrete nouns (e.g., by considering whether oneself owns or would use the named object) eliminates the effect, which is not the case for trait adjectives (Maki &McCaul, 1985: Experiment 1; Mueller et al., 1988; Symons & Johnson, 1997).^1^ An eSRE that has trait adjectives but not concrete nouns may be because the latter are not elaboratively integrated into an adult’s self-schema in the same way as the former (Maki & McCaul., 1985; Symons & Johnson, 1997). Importantly, these results suggest the eSRE magnitude varies depending on word type. If spontaneous evaluative self-referencing produces the iSRE, would eSRE findings for trait adjectives and concrete nouns replicate in the iSRE? Alternatively, if perceptual processing of target stimuli in spatio-temporal relation to a self-cue underpins the iSRE, varying the semantic properties of target stimuli should not affect iSRE presence/magnitude. Moreover, an iSRE should also be found with target stimuli that contain no semantic properties, such as pseudowords.

To investigate whether spontaneous evaluative or enhanced perceptual processing of target stimuli in relation to a self-cue produces the iSRE, we conducted an initial study examining source memory for self-paired versus stranger-paired words across different word types. Results showed better source memory for self-paired trait adjectives and concrete nouns but not pseudowords, suggesting that perceptual processing of target stimuli in relation to a self-cue does not underpin the iSRE.^2^ Notably, unlike extant eSRE findings (Maki &McCaul, 1985: Experiment 1; Mueller et al., 1988), the iSRE magnitude did not differ between trait adjectives and concrete nouns. Together, these findings indicate that it is unlikely evaluative self-referencing, even spontaneously, underpins the iSRE. Instead, incidental self-referencing likely spontaneously enhances the semantic processing of target stimuli that co-occur with a self-cue.

Critically, however, our initial study only compared source memory for words incidentally encoded to the self and a stranger. Therefore, it was unclear whether the observed iSRE for trait adjectives and concrete nouns was due to self-specific processing or familiarity-based processing as the self versus stranger comparison is inherently confounded by familiarity. Interestingly, addressing this confound, a recent study (Kim & Philipps, 2025) demonstrated that the item memory iSRE is self-specific. In this study, trait adjectives were presented with the name of oneself (self, highly familiar), a personally close other (i.e., best friend; non-self, highly familiar), or an unknown person (non-self, unfamiliar). Item recognition was better for self-paired than for friend-paired words, which was in turn better recognized than stranger-paired words, indicating that self-relevance contributes to the iSRE above and beyond familiarity.

Building on the findings of our initial study and that of Kim and Philipps (2025), we conducted the present experiment to directly test whether spontaneous semantic self-referencing underpins the iSRE. To this end, we modified Turk et al.’s (2008) incidental SRE task and had participants encode either trait adjectives, concrete nouns, or pseudowords appearing alongside their own name, their best friend’s name, or a stranger’s name. Their source memory for the words was subsequently probed in a one-step source memory test. We hypothesized to observe an iSRE relative to both the stranger-referent and the highly familiar, friend-referent, replicating the self-specificity of the iSRE (Kim & Philipps, 2025). More importantly, regarding word type, we hypothesized three different patterns of results. First, if spontaneous evaluative self-referencing drives the iSRE, we should observe an iSRE for trait adjectives (Kim et al. 2019), a weaker or absent iSRE for concrete nouns, and no iSRE for pseudowords, with the largest effect for trait adjectives. Second, if spontaneous semantic processing drives the iSRE, then a comparable iSRE should be observed for trait adjectives and concrete nouns, and an absent iSRE for pseudowords. Third, if perceptual processing of target stimuli in spatio-temporal relation to self-cues produces the iSRE, trait adjectives, concrete nouns, and pseudowords should yield iSREs of similar magnitude.

## Method

### Participants and design

An a priori power analysis using G*Power (Faul et al., 2007) for a repeated-measures, within-between interaction determined that a minimum of 183 participants (61 participants per condition) were needed based on the effect size from Kim and Philipps (2025) for the contrast between self versus friend, Cohen’s *f* =.12 (Cohen’s *d* = 0.25) at an alpha level of.05 (two-tailed) with 90% power. 255 undergraduate students from the University of Queensland participated for partial course credit. All had normal or corrected-to-normal vision. Forty-nine participants were removed from subsequent analyses because they performed poorly on the encoding task (< 50 % accuracy in target location judgments; *n* = 9), responding to < 95% of memory test trials in < 150 ms (*n* = 36), or a combination of both (*n* = 4). The final sample included 206 participants (concrete noun *n* = 69, trait adjective *n* = 69, pseudoword *n* = 68; female *n =* 139, male *n* = 65, other *n* = 2; *M*_age_ = 19.65 years, *SD* = 3.32).

The experiment had a 3 (Referent Cue: self, friend or stranger) × 3 (Word Type: trait adjectives, concrete nouns, or pseudoword) mixed factorial design, with Word Type as a between-subjects factor. Participants were randomly assigned to Word Type conditions. The study was approved by the University of Queensland Health and Behavioural Sciences, Low and Negligible Risk Ethics Sub-Committee, in accordance with the ethical standards of the 1964 Declaration of Helsinki. Participants gave informed consent prior to the start of the experiment.

### Apparatus and stimuli

Participants completed the experiment online using Pavlovia (Peirce et al., 2019) on their personal computer. For each word type condition, there were 80 words that were split into four lists of 20 words. Each participant received one list for self-trials, one list for friend-trials, another for stranger-trials, with the final list being used as foil-words in the source memory test. Lists were counterbalanced across participants. Half the words in each referent cue condition were presented on the left or right side of the referent-cue name. Trait adjectives (e.g., optimistic, energetic) were sourced from Anderson (1968), concrete nouns (e.g., hairbrush, magazine) from SUBTLEX-US (Brysbaert & New, 2009), and pseudowords (e.g., dissleit, imbitatop) were generated using a pseudoword generator, Wuggy (Keuleers & Brysbaert, 2010). Trait adjectives were matched on Anderson’s (1968) likeability ratings. Trait adjectives and concrete nouns were matched on valence using Warriner et al.’s (2013) valence ratings and on frequency using Van Heuven et al.’s (2014) Zipf scale. Trait adjectives, concrete nouns, and pseudowords were matched on character and syllabic length.

For referent-cues, participants’ preferred first names were used as the self-cue. For the friend-cue, we used the preferred name of each participant’s best friend that the participant supplied before the experiment began. For the stranger-cue, we used a gender-neutral first name, ‘Sam’. Participants were told Sam was a quiet person who enjoyed spending time outside in nature.

### Procedure

#### Encoding task

**Fig. 1:**
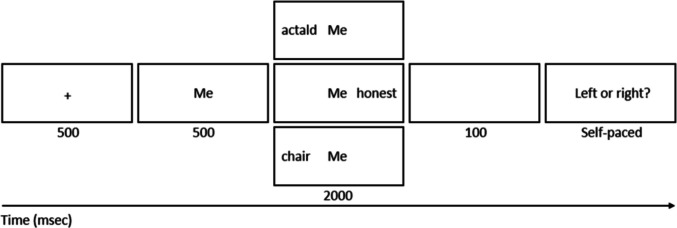
Example trial of the encoding task. “Me” was replaced with the participant’s preferred name (self-cue), the friend’s preferred name (friend-cue) and “Sam” (stranger-cue)

Each encoding trial (see Fig. 1) started with a 500-ms fixation cross. Then, a referent cue (the self-cue, the friend-cue, or the stranger-cue) appeared and remained on the screen for 2,500 ms. A target word (a trait adjective, concrete noun, or pseudoword) appeared 500 ms after referent-cue onset, on the right or left side, for 2,000 ms. That is, target word and referent cue appeared on the screen together for 2,000 ms. Following this, a blank screen appeared for 100 ms. Next, a question appeared in the centre of the screen asking if the word appeared on the left or right side of the referent cue. Participants responded by pressing the ‘O’ (left) or ‘P’ (right) key. After the key press, a blank screen appeared for 1,000 ms, followed by the next trial. Participants completed 60 (20 self-trials, 20 friend-trials, 20 stranger-trials) trials and the order of trials was randomised for each participant. Three practice trials were completed initially to ensure instructions were understood. Practice trials did not advance until the correct location response was given.

#### One-step source memory test

Following the encoding task, participants had a 1-min break before starting the surprise one-step source memory test. All four word lists of the total 80 words (20 from each referent condition and 20 foils) were presented individually in the centre of the screen in a randomised, self-paced order. Participants indicated by a button press if the word was presented during the encoding task with either their name (‘H’; self-paired), their friend’s name (‘J’; friend-paired), Sam’s name (‘K’; stranger-paired), or was a new unseen foil item (‘L’; new). If participants recognised a word from the encoding phase but were unsure with whom it was shown, they were instructed to take their best guess.

### Methodological changes from initial experiment

In our initial experiment, reported in the Online Supplementary Materials, an attention check was included immediately following the completion of the encoding task, in which participants were prompted to press the ‘Y’ key to continue. While any key press advanced past the attention check, only the ‘Y’ key was scored as correct. Additionally, a memory anticipation question was presented immediately following the source memory test, asking participants to indicate the degree to which they expected a memory test on a 7-point Likert scale (1 = strongly disagree, 7 = strongly agree).

Both of these measures were omitted from the current experiment for two reasons. First, excluding participants from the initial experiment who responded at least 5 or higher on the memory anticipation measure, failed the attention check, or both did not alter the main findings. Second, other SRE projects from our research group have shown that neither measure is a reliable predictor of participants’ data quality. Consequently, we did not consider them further in the current experiment.

### Statistical analyses

Note that although we used a classical frequentist framework for our power analysis, all reported analyses use a Bayesian framework to quantify the strength of evidence for the presence or absence of any effects of interest.^3^ For discussions of the different inferential outcomes of frequentist and Bayesian frameworks, see Dienes (2008) and Lakens (2022).

The Bayes factor (BF) provides an estimate of the likelihood of one hypothesis (e.g., the alternative hypothesis (H_1_)) over another hypothesis (e.g., the null hypothesis (H_0_)) given the observed data (Wagenmakers, Love et al., 2018a, Wagenmakers, Marsman et al., 2018b). BF_10_ expresses the relative likelihood of H_1_ over H_0_, while its reverse, BF_01_, expresses the likelihood of H_0_ over H_1_. For example, a BF_10_ value of x indicates that the observed data are x times more likely under H_1_ than under H_0_. For interpretation of BFs, we used the following conventional, rule-of-thumb classification scheme (Jeffreys, 1961; Kass & Raftery, 1995) as a reference point: No evidence when BF = 1 (i.e., the observed data are equally likely under H_0_ and H_1_), ‘weak/anecdotal’ evidence when 1 < BF ≤ 3, ‘substantial’ evidence when 3 < BF ≤ 10, ‘strong’ evidence when 10 < BF ≤ 30, ‘very strong’ evidence when 30 < BF ≤ 100, and ‘decisive’ evidence when BF > 100.

We conducted Bayesian analyses using JASP (JASP Team, 2025, ver. 0.95.3), with its default ‘objective’ priors (for *t*-tests: Cauchy distribution scaling factor *r* = 0.707; for analyses of variance (ANOVAs): *r* = 0.5, 1, and 0.354 for fixed effects, random effects, and covariates, respectively; Rouder et al., 2009, 2017; Wagenmakers, Love et al., 2018a). For ANOVAs, we set the number of samples to 500,000 to reduce Monte Carlo sampling error. For each ANOVA result, we first report the most preferred model (i.e., the model with the highest posterior model probability relative to the intercept-only null model) to emerge from a given analysis. We then report the BF_Inclusion_ value for each factor in the model (i.e., a main effect or an interaction effect), which indicates the likelihood of the data under models that included a given factor relative to matched models without the factor. For each paired and independent-samples *t*-test, we first report the BF_10_ value to indicate the likelihood of H_1_ over H_0_, followed by *δ* (median posterior distribution) for the effect size with a 95% credible interval (CI) (Kelter, 2020; Malone & Coyne, 2025; Van Doorn et al., 2021).

Self-, friend- and stranger-referent source corrected hit rate (CHR) scores were calculated individually. The self-referent source CHR score was calculated by subtracting the self-false-alarm rate proportion (proportion of new words incorrectly identified as self-words) from the proportion of self-words correctly chosen as self-words. The friend-referent source CHR score was calculated by subtracting the friend-false-alarm rate proportion from the proportion of friend-words correctly chosen as friend-words. The stranger-referent source CHR score was calculated by subtracting the stranger-false-alarm rate proportion from the proportion of stranger-words correctly chosen as stranger-words. Therefore, a CHR of 1 indicated perfect performance and 0 indicated guessing. Any one-step source memory trial responses shorter than 150 ms from stimulus onset were removed from the analysis (eliminating a total of 0.02% of responses).

## Results

**Fig. 2:**
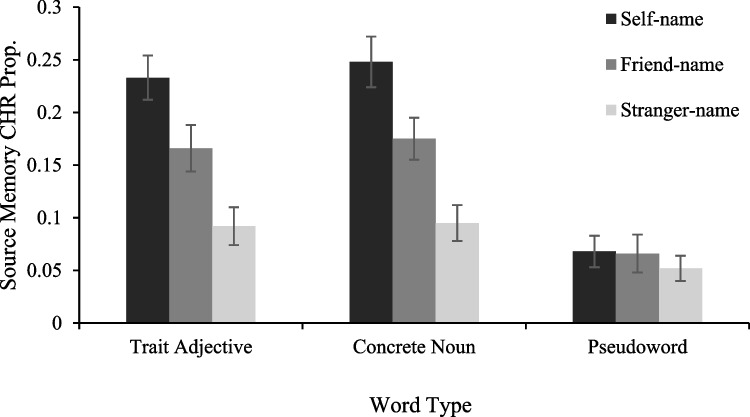
Source memory performance as a function of Referent Cue and Word Type. Error bars represent one standard error of the mean

Table 1 presents hit and false-alarm rates. The source CHRs were submitted to a 3 (Referent: self, friend or stranger) × 3 (Word Type: trait adjectives, concrete nouns, or pseudowords) mixed-model Bayesian ANOVA. The most preferred model, decisively favoured over the intercept-only null model (BF_10_ = 9.014 × 10^18^, ± 1.828%), included the main effects of Referent and Word Type, as well as the Referent × Word Type interaction. There was decisive evidence for the inclusion of both the main effect of Referent, BF_Inclusion_ = 1.006 × 10^11^, and Word Type, BF_Inclusion_ = 595456.7. Follow-up tests on Referent showed decisive evidence that source memory for self-paired words (*M* =.18, *SD* =.16) was better than friend-paired (*M* =.14, *SD* =.17), BF_10_ = 118.50, *δ* =.27, 95% CI [0.13–0.41], and stranger-paired words (*M* =.08, *SD* =.13), BF_10_ = 3.601 × 10^10^, *δ* =.54, 95% CI [0.40–0.69]. Source memory for friend-paired words was also better than stranger-paired words, BF_10_ = 357.4,* δ* =.29, 95% CI [0.15–0.]. Follow-up tests on Word Type showed decisive evidence that source memory for trait adjectives (*M* =.16, *SD* =.17) and concrete nouns (*M* =.17, *SD* =.17) was better than that of pseudowords (*M* =.06, *SD* =.12), BF_10_ = 1.990 × 10^8^, *δ* =.92, 95% CI [0.56–1.28], and BF_10_ = 2.831 × 10^9^, *δ* =.97, 95% CI [0.61–1.33], respectively, with substantial evidence that source memory for trait adjectives and concrete nouns did not differ, BF_10_ =.12, *δ* =.06, 95% CI [−0.26–0.38]. There was also decisive evidence for the inclusion of the Referent × Word Type interaction, BF_Inclusion_ = 158.8 (see Fig. 2).

**Table 1:** Mean proportion (standard error) of hits and false alarms (FAs) for source memory

	Self-name		Friend-name		Stranger-name	
	Source hit	Source FA	Source hit	Source FA	Source hit	Source FA
Concrete noun	.35 (.02)	.10 (.01)	.33 (.02)	.15 (.01)	.27 (.01)	.17 (.01)
Trait adjective	.35 (.02)	.12 (.01)	.33 (.02)	.16 (.01)	.27 (.02)	.17 (.01)
Pseudoword	.22 (.01)	.15 (.01)	.25 (.01)	.18 (.01)	.22 (.01)	.17 (.01)

Follow-up tests on the Referent × Word Type interaction using Bayesian paired-samples *t*-tests showed that source memory for trait adjectives was better for self- (*M* =.23, *SD* =.17) than friend- (*M* =.17, *SD* =.18), BF_10_ = 10.17, *δ* =.36, 95% CI [0.12–0.60], and stranger-paired words (*M* =.09, *SD* =.15), BF_10_ = 256368, *δ* =.72, 95% CI [0.45–0.98]. Source memory was also better for friend- than stranger-paired words, BF_10_ = 10.98, *δ* =.36, 95% CI [0.12–0.60]. Similarly, for concrete nouns, source memory was better for self- (*M* =.25, *SD* =.2) than friend- (*M* =.18, *SD* =.17), BF_10_ = 23.79, *δ* =.39, 95% CI [0.15–0.64], and stranger-paired words (*M* =.1, *SD* =.14), BF_10_ = 5.456 × 10^6^, *δ* =.81, 95% CI [0.54–1.08]. Again, source memory was better for friend- than stranger-paired words, BF_10_ = 19.64, *δ* =.39, 95% CI [0.15–0.63]. In contrast, for pseudowords, source memory for self-paired words (*M* =.07, *SD* =.12) did not differ with friend-paired words (*M* =.07, *SD* =.15), BF_10_ = 134,* δ* =.01, 95% CI [−0.22–0.24], or stranger-paired words (*M* =.05, *SD* =.1), BF_10_ =.184, *δ* =.01, 95% CI [−0.14–0.33]. Source memory for friend-paired and stranger-paired words also did not differ, BF_10_ =.165, *δ* =.07, 95% CI [−0.15–0.31].

Finally, to investigate whether the iSRE magnitude differed between trait adjectives and concrete nouns, we submitted the source memory CHRs to a 3 (Referent: self, friend or stranger) × 2 (Word Type: trait adjectives or concrete nouns) mixed-model Bayesian ANOVA. The most preferred model, decisively favoured over the intercept-only null model (BF_10_ = 7.787 × 10^13^, ±.741%) included only the main effect of Referent. There was decisive evidence for the inclusion of the main effect of, Referent, BF_Inclusion_ = 8.017 × 10^13^. Critically, there was very strong evidence against the inclusion of the Referent × Word Type interaction, BF_Inclusion_ =.050. Therefore, the iSRE magnitude did not differ between trait adjectives and concrete nouns.

## Discussion

The current study investigated if the iSRE arises from spontaneous evaluative self-referencing, spontaneous semantic self-referencing, or perceptually processing target stimuli in relation to a self-cue, by using words with varying semantic properties (trait adjectives, concrete nouns, or pseudowords). Supporting our hypotheses that the iSRE involves spontaneous semantic self-referencing, iSRE magnitude in source memory varied systematically by word type and referent: We found iSREs for self-friend and self-stranger comparisons with trait adjectives and concrete nouns but not pseudowords, confirming that spontaneous, preferential attention to a self-cue is insufficient to support source memory when the opportunity for semantic processing is removed. However, contrary to our spontaneous evaluative self-referencing hypothesis, robust iSREs of similar magnitude were found for trait adjectives and concrete nouns. Thus, the iSRE may draw on a relatively basic semantic connection between the self and a concurrently presented stimulus, rather than spontaneous *evaluation* of the word as self-referent. Finally, our findings of the iSRE for self-friend and self-stranger comparisons with semantically meaningful words extend Kim and Philipps’ (2025) findings from item memory to source memory and provide further evidence that the iSRE is underpinned by spontaneous self-referencing rather than familiarity-related processes alone.

Our findings suggest that perceptual processing of target stimuli in spatio-temporal relation to a self-cue alone cannot produce an iSRE, since the iSRE was absent for pseudowords. Therefore, it is unlikely the iSRE arises only from automatic attentional processes (Kim et al., 2018). Instead, the semantic relationship between the stimulus’ meaning and the self may be processed consciously, subsequently improving source memory for self-paired words. Of note, however, the similar iSRE magnitude across trait adjectives and concrete nouns, as opposed to a greater eSRE for trait adjectives versus concrete nouns (Maki & McCaul 1985: Experiment 1; Mueller et al., 1988; Symons & Johnson, 1997), suggests qualitative differences between how spontaneous semantic and task-directed evaluative self-referencing affects source memory. Although these early eSRE findings would benefit from replication and extension to source memory, we speculate these differences may be due to the types of semantic processing engaged by spontaneous semantic and task-directed evaluative self-referencing.

The eSRE is based on both semantic elaboration and organisation (Klein, 2012; Symons & Johnson, 1997). Semantic elaboration creates enhanced associations between incoming stimuli and semantic/episodic memories contained within one’s autobiographical self-knowledge base, while semantic organisation enhances associations between the incoming stimuli themselves within the encoding context (e.g., ‘me’ vs. ‘not me’ categories; Klein, 2012). The finding that the eSRE increases with age, reflecting an expanding autobiographical and self-knowledge base, while the iSRE remains developmentally stable, suggests that, unlike the eSRE, the iSRE may not rely on elaborating incoming stimuli with long-term semantic/episodic memories (Hutchison et al., 2021). Thus, semantic elaboration may be the key mechanism differentiating the self-memory advantages produced by task-directed evaluative versus non-task-directed spontaneous semantic self-referencing. This account may explain why the iSRE (current study) but not the eSRE (Maki & McCaul, 1985: Experiment 1; Mueller et al., 1988) presents similarly across trait adjectives and concrete nouns. Task-directed evaluative self-referencing (eSRE) may elicit stronger semantic elaboration for trait adjectives than concrete nouns because traits are more readily integrated with the young adult self-schema (Maki & McCaul, 1985; Symons & Johnson, 1997). In contrast, if spontaneous semantic self-referencing (iSRE) does not involve semantic elaboration, such that incoming stimuli are not evaluated against stored self-knowledge, the trait-noun difference observed with the eSRE (Maki & McCaul, 1985: Experiment 1; Mueller et al., 1988) should not arise with the iSRE.

It is interesting to note that the mere ownership effect (MOE; Cunningham et al., 2008), which, like the iSRE, is driven by non-evaluative, task-directed self-stimuli associations, is underpinned by semantic organisation rather than semantic elaboration (Englert & Wentura, 2016). Englert and Wentura provided tentative evidence against a role of semantic elaboration in the MOE by showing that the time it took the participants to assess the semantic meaning of an object in a simple object-label matching task did not differ for self-owned versus other-owned objects. In contrast, strong evidence for the role of semantic organisation in the MOE comes from their finding that the MOE was eliminated when self versus other encoding trials were preceded by a semantic categorisation task (“is this object real or artificial?”), but not a perceptual categorisation task (“is this object green or purple?”). Therefore suggesting the MOE occurs only when an alternative, strong semantic organizational principle is not already in place. Although both the iSRE and MOE arise in the absence of task-directed self-appraisals, it remains an open question whether the iSRE is similarly underpinned by semantic organization.

The present findings fit neatly within the broader literature on self-bias effects suggesting that self-processing does not occur automatically (Englert & Wentura, 2016; Falbén et al., 2019; Kim et al., 2018; Schäfer et al., 2015, 2020). Our finding of an iSRE with semantically meaningful words but not pseudowords suggests stimuli do not automatically receive enhanced processing merely because they co-occur with a self-cue. Instead, stimuli may first need to be identified as self-relevant by association with concepts contained in self-knowledge. In this regard, the current study corresponds with prior work showing that the prioritized status of self- relative to other-associated stimuli in information processing (i.e., the self-prioritization effect; Sui et al., 2012) requires semantic appraisal of the stimuli (Falbén et al., 2019) and arises at a post-perceptual, conceptual level (Schäfer et al., 2015). Once bound to the self-concept, self-relevant stimuli, relative to other-relevant stimuli, may benefit from stronger associations within long-term memory, allowing for enhanced attention, perception and/or memory processing. In this sense, self-relevance acts as an ‘associative glue’, binding newly identified self-relevant information with pre-existing self-relevant knowledge (Schӓfer et al., 2020; Sui & Humphreys, 2015). Through associative learning, self-relevant connections may be continuously reinforced, ultimately becoming more salient and strongly integrated into long-term memory than non-self-relevant connections (Schӓfer et al., 2020).

In the present study, we used pseudowords precisely because they lack semantic meaning, allowing us to investigate whether automatic, preferential attention to stimuli, without additional processing from semantic knowledge, produces the iSRE. This approach, however, raises the question of how target stimulus’ familiarity and meaningfulness contribute to iSRE presence/magnitude, given that pseudowords are inherently less familiar and meaningful than real words. Although beyond the scope of the present study, exploring how target stimulus’ familiarity and meaningfulness influence the manifestation of the iSRE remains an important question for future research.

We also included a high-familiarity other referent, one’s best friend, to determine whether spontaneous self-specific or familiarity-processing underpinned the iSRE. Our findings of an iSRE with real words for the self versus friend and self versus stranger comparisons show that self-processing contributes to the self-memory advantage over and beyond familiarity-processing. However, an incidental friend-memory advantage was also present for friend versus stranger comparisons with real words, suggesting that familiarity-processing does contribute to the iSRE, albeit to a lesser extent than self-processing. Had self-specific processing alone underpinned the iSRE, we would have observed an incidental self-memory advantage in the absence of an incidental friend-memory advantage. Therefore, familiarity processes underpinning the iSRE to some extent cannot be conclusively ruled out.

Although iSRE tasks do not require participants to semantically process target stimuli in reference to the self, our findings suggest semantic self-referencing occurs spontaneously. According to Johnson et al.’s (1993) source-monitoring framework, episodic memory for material improves when encoding and binding are enhanced because the material may be useful later. Therefore, in contexts akin to everyday life, the self-memory system may spontaneously prioritise information that could subsequently have self-relevance (Cunningham et al., 2013; Turk et al., 2008). Consistent with this view, research on mind-wandering shows that task-unrelated thoughts are often self-referential (for a review, see D’Argembeau, 2018), and neuroimaging findings reveal substantial overlap between the default network (i.e., the brain regions that are more active during rest than during active tasks) and brain regions engaged during self-related tasks, such as the medial prefrontal cortex and anterior cingulate cortex. Spontaneous self-referential thought at both the behavioural and the neural levels may serve several adaptive functions, such as future planning and problem-solving, as well as construction and maintenance of memories contained within the self-concept.

In summary, we found that spontaneous semantic self-referencing rather than either spontaneous evaluative self-referencing or perceptual spatio-temporal processing between the self-cue and target items produces the iSRE. Our findings suggest that the self is consistently semantically processing and integrating new information in reference to the self-schema, even in the absence of task demands to engage in such self-referential processing. Importantly, however, semantic knowledge of the information is necessary to produce an incidental self-memory advantage.

## Supplementary Information

Below is the link to the electronic supplementary material.

Supplementary file1 (32.9 KB)

## Data Availability

The datasets generated and analysed during the current study are available in the Open Science Framework repository at (https://osf.io/cta8u/). The materials and program code used in the current study are available upon reasonable request.

## Footnotes

1 We have not focussed on other eSRE concrete noun self-other comparison studies that used imagery instructions (e.g., Brown et al., 1986; Lord, 1980, 1987; Maki & McCaul, 1985: Experiment 2). We cite only Maki and McCaul (1985: Experiment 1) and Mueller et al. (1988) because their eSRE concrete noun-encoding task required dichotomous yes/no responses, paralleling the dichotomous location judgments used in iSRE tasks. Although direct eSRE versus iSRE task comparisons are outside the scope of the current study, we considered it prudent to reference eSRE concrete noun studies with comparable response requirements since we are investigating whether spontaneous evaluative self-referencing underpins the iSRE.

2 For completeness and transparency, we report the method and results of this initial study in the Online Supplementary Materials.

3 Conventional frequentist analyses yielded the same pattern of results as the Bayesian findings reported here. See the Online Supplementary Materials for the frequentist results.

## Code availability

The program code used for data analysis in the current study is available upon reasonable request.
