# Supplementary material for: Semantic knowledge of words is necessary to produce an incidental self-reference effect
Source: Psychon Bull Rev. 2026 Mar 26;33(4):121. doi: 10.3758/s13423-026-02901-y (PMC13021704; doi:10.3758/s13423-026-02901-y)
Supplement: Supplementary file 1 — Supplementary file1 (32.9 KB) [file 13423_2026_2901_MOESM1_ESM.docx]

Supplementary Materials

Initial self vs. stranger experiment methodology

**Participants and design**

An a priori power analysis using G*Power (Faul et al., 2007) determined that a minimum of 43 participants per condition were needed based on the effect size from Turk et al. (2008), Cohen’s *dz* = 0.44 for self vs. other within-subjects comparisons at an alpha level of .05 (two-tailed) with 80% power. One hundred and fifty undergraduate students (female *n* *=* 111*,* male *n* = 35, other *n* = 4; *M* age = 19.72 years, *SD* = 3.36) from the University of Queensland participated for partial course credit. All participants had normal or corrected to normal vision. The experiment had a 2 (Referent Cue: self or other) x 3 (Word Type: trait adjectives, concrete nouns, or pseudoword) mixed factorial design, with Word Type as a between-subjects factor.

Twenty-five additional participants were removed from subsequent analyses because they performed poorly on the encoding task (below 50% accuracy in target location judgments; *n* = 9), responding to < 95% of memory test trials in > 150ms (*n* = 13), or a combination of both (*n* = 3). The final sample included 150 participants (concrete noun *n* = 50, trait adjective *n* = 50, and pseudoword *n* = 50).

**Apparatus and Stimuli**

Participants completed the experiment online using Pavlovia (Peirce et al., 2019) on a laptop or desktop PC. Seventy-eight words appeared (black font on white background) during the encoding and surprise one-step source memory test. They were split into three lists of 26 words. Each participant received one list for self-trials, another for other-trials, with the final list being used as foil words in the source memory test. Lists were counterbalanced across participants. Half the words in each referent cue condition were presented on the left or right side of the referent-cue name. Trait adjectives (e.g., optimistic, energetic) were sourced from Anderson (1968), concrete nouns (e.g., hairbrush, magazine) from SUBTLEXUS(Brysbaert & New, 2009), and pseudowords (e.g., dissleit, imbitatop) were generated using a pseudoword generator, Wuggy (Keuleers & Brysbaert, 2010). Trait adjectives were matched on Anderson’s (1968) likeability ratings. Trait adjectives and concrete nouns were matched on valence using Warriner et al.’s (2013) valence ratings and on frequency using Van Heuven et al.’s (2014) Zipf scale. Trait adjectives, concrete nouns, and pseudowords were matched on character and syllabic length. Participants’ preferred first names were used as the self-cue, whereas a gender-neutral first name ‘Sam’ was used as the other-cue. Sam was introduced to the participants as a quiet person that enjoyed spending time outside in nature.

**Procedure**

**Encoding task.** Each encoding trial (see Figure 1) started with a black fixation cross in the centre of the screen (500ms). The participant’s name (the self-cue) or Sam’s name (the other-cue) appeared and remained on the screen for 2500ms. Target word (a trait adjective, concrete noun, or pseudoword) was 500ms after referent cue onset, on the right or left side for 500ms. That is, target word and referent cue appeared on the screen together for 2000ms. Following this, a white blank screen appeared for 100ms. Next, a question appeared in the centre of the screen asking if the word appeared on the left or right side of the referent cue. Participants responded by pressing the ‘O’ (left) or ‘P’ (right) key. After the key press, a white blank screen appeared for 1000ms, followed by the next trial. Self- and other-cue order was randomised, as was the order of target word type (trait adjective, concrete noun, or pseudoword). Participants completed 52 (26 self-trials, 26 other-trials) trials during the encoding task. Three practice trials were completed by participants before the experimental procedure to ensure instructions were understood. Practice trials did not advance until the correct location response was given.

**Attention Check.** Participants completed an attention check immediately following completion of the encoding task *‘Please press the Y key on the keyboard to continue’* (Bentley et al., 2017). Any key press advanced the attention check, however, only pressing the ‘Y’ key was scored as correct. Pressing any other key was scored as incorrect.

**One step source memory test.** Following the attention check, participants had a one-minute break before starting the surprise one-step source memory test. All three word lists of the total 78 words (26 self-trials, 26 other-trials, and 26 foil-trials) were presented individually in black font in the centre of the screen in a randomised order. In self-paced trials, participants indicated if they thought the word was previously presented during the encoding task with either their name, Sam’s name, or was a new unseen foil item. Responses were recorded by a button press using the ‘J’ (self-paired word), ‘K’ (other-paired word), or ‘L’ (new word) key. If participants recognised a word from the encoding phase but were unsure of whom it was shown with, they were instructed to take their best guess of either themself or Sam.

**Memory test guess.** After the one-step source memory test, participants rated their agreeableness on a 7-point Likert scale ranging from 1 (*strongly disagree*) to 7 (*strongly agree*) to the following statement *‘I guessed there would be a memory test in the experiment’*. Overall, 55 participants responded at least 5 or higher on the memory anticipation measure (*n* = 46) and failed the attention check (*n* = 8), or a combination of both (*n* = 1). However, as removing these participants from subsequent analyses did not alter the main findings, their data were retained in the analyses.

**Statistical Analyses.** All statistical tests used the .05 level of significance. Effect sizes are reported as partial eta squared (*η_p_^2^*) for analyses of variance (ANOVAs) and Cohen’s *d* for paired-samples t-tests. All reported *p* values for pair-wise comparisons were Holm corrected. Note that as in the manuscript, although we used a classical frequentist framework for our power analysis, all reported analyses use a Bayesian framework to quantify the strength of evidence for the presence or absence of any effects of interest.

Initial self vs. stranger experiment results.

The source CHRs were submitted to a 2 (Referent: self or stranger) x 3 (Word Type: trait adjectives, concrete nouns or pseudowords) mixed factorial ANOVA. There was a significant main effect of Referent, *F*(1, 147) = 61.50, *p* < .001, *η_p_^2^* = .30, BF_Inclusion_ = 1.1983 x 10^10^. Source memory for self-paired words (*M* = .20, *SD* = .16) was better than stranger-paired (*M* = .08, *SD* = .14). There was also a significant main effect of Word Type, *F*(2, 147) = 10.48, *p* < .001, *η_p_^2^* = .13, BF_Inclusion_ = 290.798. Follow up independent-samples t-tests on Word Type showed that source memory for trait adjectives (*M* = .16, *SD* = .15) and concrete nouns (*M* = .18, SD = .17) was better than that of pseudowords (*M* = .08, *SD* = .13), *t*(147) = 3.39, *p* = .002, *d* = .53, BF_10_ = 64.683, *δ* = .71, 95% CI [0.31 – 1.11] and *t*(147) = 4.36, *p* < .001, *d* = .68, BF_10_ = 2118.414, *δ* = .80, 95% CI [0.39 – 1.21], respectively. Source memory for trait adjectives and concrete nouns did not differ, *t*(147) = .97, *p* = .335, *d* = .15, BF_10_ = .226, *δ* = .16, 95% CI [-0.21 – 0.53]. The Referent x Word Type interaction was also significant, *F*(2, 147) = 10.48, *p* < .001, η_p_^2^ = .13, BF_Inclusion_ = 5.548.

**Table 1.** Mean proportion (standard error) of hits and false alarms (FA) for source memory as a function of Referent and Word Type.

|  | Self-name | | Stranger-name | |
| --- | --- | --- | --- | --- |
|  | Source hit | Source FA | Source hit | Source FA |
| Concrete Noun | .41 (.02) | .15 (.02) | .29 (.02) | .18 (.02) |
| Trait Adjective | .45 (.02) | .21 (.02) | .31 (.02) | .24 (.02) |
| Pseudoword | .31 (.02) | .20 (.02) | .27 (.02) | .22 (.02) |

Planned pairwise comparisons using paired-samples t-tests showed that source memory for trait adjectives was better for self- (*M* = .24, *SD* = .17) than stranger-paired words (*M* = .07, *SD* = .14), *t*(147) = 6.32, *p* < .001, *d* = 1.11, BF_10_ = 27469, *δ* = .78, 95% CI [0.46 – 1.10]. Similarly, for concrete nouns, source memory was better for self- (*M* = .25, *SD* = .17) than stranger-paired words (*M* = .11, *SD* = .16), *t*(147) = 5.25, *p* < .001, *d* = .93, BF_10_ = 13731, *δ* = .75, 95% CI [0.44 – 1.07]. In contrast, for pseudowords, source memory for self-paired words (*M* = .11, *SD* = .13) did not differ with stranger-paired words (*M* = .05, *SD* = .14), *t*(147) = 2.02, *p* = .289, *d* = .36, BF_10_ = 1.231, *δ* = .28, 95% CI [0.01 – 0.56].

Finally, to investigate whether the iSRE magnitude differed between trait adjectives and concrete nouns, we submitted the source memory CHRs to a 2 (Referent: self or stranger) x 2 (Word Type: trait adjectives or concrete nouns) mixed factorial ANOVA. Critically, the Referent x Word Type interaction was not significant, *F*(1, 98) = .54, *p* = .464, *η_p_^2^* = .01, BF_Inclusion_ = .268. Therefore, the iSRE magnitude did not differ between trait adjectives and concrete nouns.

**Figure 1.** Source memory performance as a function of Referent and Word Type. Error bars represent one standard error of the mean.

Self vs. friend vs. stranger results frequentist statistics

The source CHRs were submitted to a 3 (Referent: self, friend or stranger) x 3 (Word Type: trait adjectives, concrete nouns or pseudowords) mixed factorial ANOVA. There was a significant main effect of Referent, *F*(2, 406) = 33.90, *p* < .001, *η_p_^2^* = .14. Follow up paired samples t-tests showed that source memory for self-paired words (*M* = .18, *SD* = .16) was better than friend-paired (*M* = .14, *SD* = .17), *t* = 3.96, *p* < .001, *d* = .3, and stranger-paired words (*M* = .08 *SD* = .13), *t* = 8.28, *p* < .001, *d* = .66. Source memory for friend-paired words was also better than stranger-paired words, *t* = 4.24, *p* < .001, *d* = .36. Next, there was a significant main effect of Word Type, *F*(2, 203) = 19.44, *p* < .001, *η_p_^2^* = .16. Follow up independent-samples t-tests on Word Type showed that source memory for trait adjectives (*M* = .16, *SD* = .17) and concrete nouns (*M* = .17, SD = .17) was better than that of pseudowords (*M* = .06, *SD* = .12), *t* = 5.17, *p* < .001, *d* = .66, and *t* = 5.61, *p* < .001, *d* = .71, respectively. Source memory for trait adjectives and concrete nouns did not differ, *t* = .44, *p* = .660, *d* = .06. The Referent x Word Type interaction was also significant, *F*(4, 406) = 6.04, *p* < .001, η_p_^2^ = .06.

Planned pairwise comparisons using paired-samples t-tests showed that source memory for trait adjectives was better for self- (*M* = .23, *SD* = .17) than friend- (*M* = .17, *SD* = .18), *t*  = 3.329, *p* = .025, *d* =.43, and stranger-paired words (*M* = .09, *SD* = .15), *t*  = 6.54,  *p* < .001, *d* = .91. Source memory was also better for friend- than stranger-paired words, *t* = 3.24, *p* = .025, *d* = .48. Similarly, for concrete nouns, source memory was better for self- (*M* = .25, *SD* = .2) than friend- (*M* = .18, *SD* = .17), *t* = 3.54, *p* = .011, *d* = .47, and stranger-paired words (*M* = .1, *SD* = .14), *t* = 7.09, *p* < .001, *d* = .98. Again, source memory was better for friend- than stranger-paired words, *t* = 3.5, *p* = .012, *d* = .52. In contrast, for pseudowords, source memory for self-paired words (*M* = .07, *SD* = .12) did not differ with friend-paired (*M* = .07, *SD* = .15), *t* = .09, *p* = 1, *d* = .01, and stranger-paired words *(M* = .05, *SD* = .1), *t* = .74,  *p* = 1, *d* = .1. Source memory for friend-paired and stranger-paired words also did not differ, *t* = .62, *p* = 1, *d* = .09.

Finally, to investigate whether the iSRE magnitude differed between trait adjectives and concrete nouns, we submitted the source memory CHRs to a 3 (Referent: self, friend or stranger) x 2 (Word Type: trait adjectives or concrete nouns) mixed factorial ANOVA. Critically, the Referent x Word Type interaction was not significant, *F*(2, 272) = .07, *p* = .932, *η_p_^2^* = 0. Therefore, the iSRE magnitude did not differ between trait adjectives and concrete nouns.
